# Epidemiological status of depressive disorders in the Middle East and North Africa from 1990 to 2019

**DOI:** 10.34172/hpp.2022.39

**Published:** 2022-12-10

**Authors:** Mehdi Moradinazar, Parmida Mirzaei, Saied Moradivafa, Mahdieh Saeedi, Mona Basiri, Mohammad Shakiba

**Affiliations:** ^1^Behavioral Disease Research Center, Kermanshah University of Medical Sciences, Kermanshah, Iran; ^2^Department of Psychology, Faculty of Psychology, Kermanshah branch Islamic Azad University, Kermanshah, Iran

**Keywords:** Depressive disorders, Epidemiology, Middle East, North Africa

## Abstract

**Background:** Depressive disorders are one of the most common mental health diseases, which are associated with adverse life events such as increased risk of self-injury. This study was aimed to measure the epidemiological status and the burden of depressive disorders in the Middle East and North Africa (MENA) countries.

**Methods:** The study population included 21 countries in the MENA region, covering a population of about 600 million. The Global Burden of Disease (GBD) 2019 database was used. The disability-adjusted life years (DALYs) were estimated by the years lived with disability (YLDs) component. The prevalence, incidence, and the DALYs rates per 100000 people by age-standardized rate (ASR) were measured.

**Results:** In 2019, the highest prevalence 6198.95 (95% uncertainty interval [UI], 5402.41- 7108.39), incidence 7864.2 (95% UI, 6719.71-9216.83), and DALYs 1168.68 (95% UI, 802.95- 1624.31) per 100,000 were in Palestine by ASR. Between 1990 and 2019, the depressive disorders-related prevalence, incidence, and DALYs rates in the MENA region increased by 0.004%, 0.006%, and 0.005%. The highest increment of the prevalence, incidence, and DALYs rates was related to Saudi Arabia by 0.05%, 0.064%, and 0.055%. The highest percentage of major depressive disorder (MDD)-related DALYs attributed to intimate partner violence was related to Iran (101.1). Also, the highest percentage of MDD-related DALYs attributed to childhood sexual abuse (34.26) and bullying victimization (49.81) was related to Palestine.

**Conclusion:** Given the increasing trend of depressive disorders in MENA region, mental health programs should be prioritized across the MENA countries, with significant contributions and involvement of the governments.

## Introduction

 Depressive disorders are one of the most common mental health diseases globally, affecting about 121 million people and responsible for 5% of disability-adjusted life years (DALYs) across the world.^1^ In 2017, depressive disorders were the second leading cause of disease burden in terms of years lived with disability (YLDs) and the sixth leading cause of DALYs worldwide.^2^ According to World Health Organization (WHO), major depressive disorder (MDD) will become the leading cause of disability in 2030.^3^

 Depressive disorders are associated with adverse life events e.g. suicide, inverse health outcomes, disruptions of family relationships, reduced work performance, sedentary, increased risk of self-injury, drug abuse, and reduced life expectancy.^4-7^ Furthermore, patients with depressive disorder have increased the risks of developing chronic disease e.g. cardiovascular diseases, and receiving poor treatment and increased morbidity and mortality.^8,9^

 Health policymakers should be aware of their country’s health and compared it with other countries with the same socio-demographic Index. In the Middle East and North Africa, (MENA) most socio-demographic indexes are similar. They should also use other countries’ experiences in improving their health care system. Tailoring interventions to promote mental health requires an understanding of how mental diseases differ across countries.^10^ The Global Burden of Disease (GBD) studies estimate the incidence, prevalence, death, and DALYs of all diseases for all regions, so, it creates an opportunity to compare countries and explain the pattern of diseases.

 Therefore, the aim of this study was to determine the epidemiological features and the burden of depressive disorders in the MENA countries to develop beneficial policies for decreasing the incidence, prevalence, and consequences of depressive disorders in these regions.

## Materials and Methods

###  Geographical location and population 

 We selected two regions including the MENA. MENA region, with 21 countries consisting of Afghanistan, Algeria, Bahrain, Egypt, Iran, Iraq, Jordan, Kuwait, Lebanon, Libya, Morocco, Palestine Oman, Qatar, Saudi Arabia, Sudan, Syria, Tunisia, Turkey, United Arab Emirates (UAE), and Yemen, covers a population of approximately 600 million.

###  Data collection and quality control

 We used the latest data refresh from the GBD (the 2019 update) in the current study. This dataset contains annual reports from 1990 to 2019 for depressive disorders in all countries and regions. The data of GBD estimate the incidence, prevalence, mortality, and DALYs of 369 diseases and injuries, and 84 risk factors in terms of age, sex, year, location, income group in 204 countries and regions from 1990 to 2019 (https://vizhub.healthdata.org/).

 The methodological details of GBD 2019 have been reported elsewhere.^11-13^ At GBD 2019, the prevalence, incidence, remission, duration, and/or excess mortality associated with depressive disorders were searched via the four stages including electronic searches of the peer-reviewed literature in PsycInfo, Embase, and PubMed databases, the grey literature, expert consultation, and institutional collaborations. In the estimation of depressive disorders, epidemiological data were generated using DisMod-MR (version 2.1), a meta-regression computational tool for Bayesian disease modelling that is the standard GBD modelling approach for describing non-fatal health outcomes by location, age, sex, and year. In the GBD system, modeling for all countries is based on the quality and accessibility of data. To make valid and correct comparisons between different countries and times, the same method was applied for each location and year in MENA region.

###  Deﬁnition of depressive, bipolar and anxiety disorders 

 At GBD 2019, depressive disorders included MDD and dysthymia. We defined depressive disorders according to the criteria proposed in the Diagnostic and Statistical Manual of Mental Disorders-IV (DSM-IV) and the International Classification of Diseases 10 (ICD-10).^14^ The DSM-IV (296.21–24, 296.31–34) defines MDD as an episodic disorder with a prolonged outcome and an increased risk of death,^15^ comparable to ICD-10’s definition of recurrent depressive disorder (F32.0–9, F33.0–9).^16^ MDD is the presence of at least one major depressive episode (i.e., an experience of depressed mood almost all day, every day, for at least 2 weeks). Dysthymia is a less severe depressed mood compared to MDD, lasting several years, with low rates of remission and no increased risk of death (DSM-IV: 300.4; ICD-10: F34.1).^15,16^ Anxiety disorders were contained the following codes ICD-10: F40-42, F43.0, F43.1, F93.0-93.2, F93.8, and DSM-IV-TR: 300.0-300.3, 208.3, 309.21, 309.81, which included panic disorder, agoraphobia, specific phobia, social phobia, obsessive-compulsive disorder, posttraumatic stress disorder, and generalized anxiety disorder.^15,16^ Bipolar disorder is characterized by the occurrence of at least one manic or mixed-manic episode during the patient’s lifetime (DSM-IV: 296.0–296.89; ICD-10: F31.0–9).^15,16^

 GBD study evaluated the different risk factors attributable to depressive disorder-related DALYs such as bullying victimization, childhood sexual abuse, and intimate partner violence.

###  DALY calculation 

 In the current study, we extracted information from the GBD network, and data on the incidence, prevalence, DALYs, and attributable risk factors for depressive disorders were analyzed. DALYs are computed by summing up the years of life lost (YLLs) and the YLDs.^17,18^ In the present study, although, the YLL component was not calculated given that depressive disorders are not a cause of death in the GBD study, according to the ICD-10 criteria for categorical designation of the cause of death to a single underlying cause.^1^ Therefore, DALYs were estimated for depressive disorders from the YLD component. YLDs are counted by the prevalence of individual sequelae of the disease multiplied by their corresponding disability weights without age weighting. The age-standardized rate (ASR), age-standardized incidence rate, age-standardized prevalence, and age-standardized DALYs rate are reported to universalize the population size and age structure, referring to rates per 100 000 people for all national locations.^19,20^

 Moreover, all data were extracted from GBD (https://vizhub.healthdata.org/). In general, the external validity of GBD was evaluated by performing cross-validation on a limited number of sequelae due to the computational time and complexity of this analysis. Then, we analyzed the data based on the study’s objectives e.g. age groups, risk factors, gender and etc. Also all estimates were reported with 95% uncertainty intervals (UIs). Moreover, all analyses and figures were applied by Microsoft Office Excel 2016.

## Results

 In 1990, the highest incidence rates of depressive disorders were in Palestine 8000.35 (95% UI, 6792.27-9391.16), Bahrain 6171.47 (95% UI, 5270.56-7195), and Tunisia 6154.56 (95% UI, 5235.42-7219.26) per 100 000, and also the countries of Egypt 4253.72 (95% UI, 3614.57-4997.06), UAE 4382.36 (95% UI, 3739.05-5148.89) and Iraq 4448.66 (95% UI, 3767.14-5232.58) per 100 000 had the lowest incidence rate, respectively. But in 2019, Palestine 7864.2 (95% UI, 6719.71-9216.83), Tunisia 6138.57 (95% UI, 5181.93-7228.23), and Yemen 5910.92 (95% UI, 4992.06-6992.41) per 100 000 continued to have the highest incidence rates of depressive disorders, and the lowest rate was in UAE 4031.52 (95% UI, 3408.6-4782.79) per 100,000. The incidence and prevalence rate of depressive disorders in the MENA region was found to be higher than the global average. Furthermore, the average global incidence rate of depressive disorders decreased from 3681.24 (95% UI, 3239.27-4150.12) to 3588.25 (95% UI, 3152.71-4060.42) between 1990 and 2019. This decreasement was also found in the MENA region.

 In 1990, the highest prevalence rates of depressive disorders by ASR were in Palestine 6296.83 (95% UI, 5462.38-7216.09), Tunisia 5053.8 (95% UI, 4413.32-5769.25), and Bahrain 5038.74 (95% UI, 4386.9-5753.18) per 100 000 and as well as, Palestine 6198.95 (95% UI, 5402.41-7108.39) and Tunisia 5049.41 (95% UI, 4363.94-5811.47) per 100 000 continued to have the highest prevalence rates in 2019. Moreover, the average global prevalence rate of depressive disorders slightly decreased from 3486.17 (95% UI, 3140.76-3855.74) to 3440.05 (95% UI, 3097.01-3817.64) between 1990 and 2019. This decreasement was also found in the MENA region.

 In 1990, the highest DALYs rate of depressive disorders were in Palestine 1189.65 (95% UI, 812.13-1673.48), Tunisia 933.97 (95% UI, 640.32-1284.62), and Bahrain 932.78 (95% UI, 644.37-1294.62) per 100 000 and as well as, Palestine 1168.68 (95% UI, 802.95-1624.31) and Tunisia 930.15 (95% UI, 636.46-1292.58) per 100 000 continued to have the highest DALYs rate in 2019. Moreover, the average global DALYs rate of depressive disorders slightly decreased from 588.57 (95% UI, 413.88-803.35) to 577.75 (95% UI, 405.79-788.88) between 1990 and 2019. This decreasement was also found in the MENA region Table 1.

**Table 1:** Comparing the burden of depressive disorders in the world and MENA from 1990 to 2019 by ASR

**Country**	**Year**	**Incidence rate of depressive disorders by ASR**	**Prevalence rate of depressive disorders by ASR**	**DALYs rate of depressive disorders by ASR**
Afghanistan	1990	6046.05 (5129.58-7144.85)	4996.12 (4355.1-5759.66)	895.23 (611.17-1248.90)
	2019	5985.11 (5093.58-7009.94)	4945.17 (4291.64-5668.8)	890.19 (608.04-1238.01)
Algeria	1990	4942.96 (4218.28-5823.45)	4252.15 (3711.94-4896.58)	763 (520.54-1056.02)
	2019	4763.39 (4043.84-5612.9)	4129.68 (3574.09-4777.56)	736.51 (500.15-1018.75)
Bahrain	1990	6171.47 (5270.56-7195)	5038.74 (4386.9-5753.18)	932.78 (644.37-1294.62)
	2019	5415.29 (4630.22-6335.16)	4528.09 (3960.69-5182.84)	823.25 (563.09-1149.98)
Egypt	1990	4253.72 (3614.57-4997.06)	3792.1 (3287.38-4387.10)	664.78 (451.87-916.04)
	2019	4487.85 (3816.95-5268.76)	3939.76 (3410.46-4500.94)	696.61 (472.23-956.27)
Global	1990	3681.24 (3239.27-4150.12)	3486.17 (3140.76-3855.74)	588.57 (413.88-803.35)
	2019	3588.25 (3152.71-4060.42)	3440.05 (3097.01-3817.64)	577.75 (405.79-788.88)
Iran	1990	5599.5 (4666.17-6625.94)	4661.58 (4064.27-5344.71)	848.12 (575.96-1182.04)
	2019	5895.48 (4929.12-6966.62)	4860.69 (4240.18-5584.82)	890.32 (605.75-1247.77)
Iraq	1990	4448.66 (3767.14-5232.58)	3922.52 (3425.92-4529.28)	686.5 (468.24-950.13)
	2019	4465.11 (3802.93-5230.58)	3932.65 (3426.45-4500.69)	690.87 (476.11-965.21)
Jordan	1990	5415.26 (4569.5-6364.34)	4553.9 (3958-5241.66)	826.33 (563.62-1136.39)
	2019	4819.98 (4084.46-5689.46)	4152.11 (3582.15-4766.43)	743.53 (505.02-1027.87)
Kuwait	1990	4492.34 (3795.81-5317.27)	3911.98 (3380.24-4526.68)	698.16 (475.93-963.98)
	2019	4677.72 (3950.1-5476.59)	4060.3 (3523.34-4676.52)	723.95 (488.4-1001.48)
Lebanon	1990	5216.69 (4434.71-6123.69)	4439.66 (3904.87-5099.63)	796.6 (543.93-1108.29)
	2019	5536.84 (4681.83-6501.7)	4652.71 (4030.42-5366.75)	842.01 (576.15-1165.86)
Libya	1990	4927.87 (4212.09-5844.03)	4226.45 (3692.69-4880.72)	759.43 (517.69-1049.98)
	2019	5183.19 (4425.91-6142.7)	4406.43 (3844.8-5095.2)	791.47 (540.3-1091.49)
Morocco	1990	6341.63 (5366.53-7452.26)	5183.35 (4521.6-5959.96)	957.83 (654.47-1339.3)
	2019	6189.88 (5278.46-7322.91)	5078.54 (4428.6-5830.34)	934.58 (640.09-1313.79)
MENA	1990	5068.92 (4380.9-5843.83)	4329.06 (3822.92-4912.06)	777 (533.94-1064.69)
	2019	5098.6 (4378.86-5947.72)	4348.89 (3807.29-4971.11)	781.06 (535.18-1075.62)
Oman	1990	4544.17 (3869.46-5328.87)	3946.9 (3447.15-4529.99)	703.26 (476.86-972.04)
	2019	4584.86 (3908.84-5373.75)	3963 (3481.35-4574)	707.8 (481.49-976.56)
Palestine	1990	8000.35 (6792.27-9391.16)	6296.83 (5462.38-7216.09)	1189.65 (812.13-1673.48)
	2019	7864.2 (6719.71-9216.83)	6198.95 (5402.41-7108.39)	1168.68 (802.95-1624.31)
Qatar	1990	4961.33 (4245.73-5808.92)	4199.02 (3674.22-4805.58)	760.15 (521.46-1044.81)
	2019	4616.48 (3941.61-5428.45)	3951.38 (3427.26-4517.38)	708.38 (487.12-981.53)
Saudi Arabia	1990	4555.99 (3872.37-5377.22)	4015.48 (3482.63-4599.7)	709.39 (482.79-984.97)
	2019	4848.4 (4127.2-5680.67)	4216.07 (3680.65-4857.07)	748.71 (515.68-1040.33)
Sudan	1990	5319.24 (4502.44-6312.38)	4505.25 (3923.83-5245.65)	815.34 (556.71-1123.54)
	2019	5201.33 (4401.68-6177.6)	4423.1 (3873.47-5103.09)	797.86 (547.88-1109.1)
Syria	1990	4737.03 (4027.84-5539.79)	4112.07 (3562.24-4745.75)	733.55 (498.93-1010.88)
	2019	4747.27 (4018.18-5598.94)	4132.86 (3574.24-4779.18)	731.25 (493.6-1010.88)
Tunisia	1990	6154.56 (5235.42-7219.26)	5053.8 (4413.32-5769.25)	933.97 (640.32-1284.62)
	2019	6138.57 (5181.93-7228.23)	5049.41 (4363.94-5811.47)	930.15 (636.46-1292.58)
Turkey	1990	4641.15 (4155.41-5153.74)	4044.07 (3649.26-4549.68)	717.21 (502.41-972.93)
	2019	4471.83 (3835.07-5226.36)	3942.27 (3418.56-4526.9)	696.29 (477.3-952.56)
United Arab Emirates	1990	4382.36 (3739.05-5148.89)	3820.06 (3337.49-4365.74)	680.05 (463.87-949.43)
	2019	4031.52 (3408.6-4782.79)	3578.49 (3093.07-4133.28)	628.63 (425.93-866.17)
Yemen	1990	5854.24 (4897.22-6980.68)	4857.03 (4202.84-5625.03)	882.96 (596.45-1230)
	2019	5910.92 (4992.06-6992.41)	4895.37 (4231.66-5645.47)	890.99 (607.6-1235.38)

**Figure 1:**
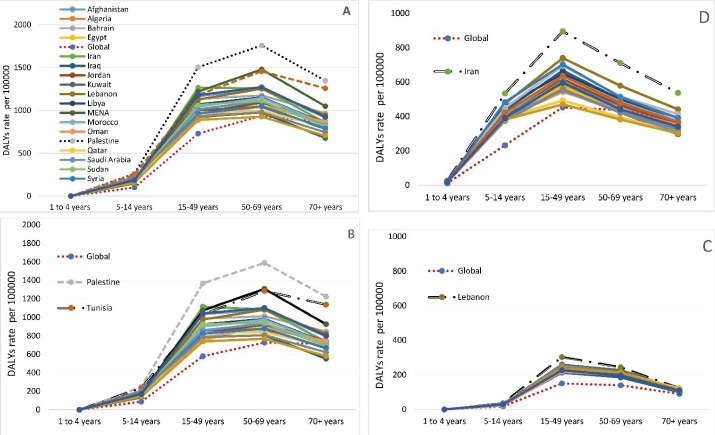
The age trends of the DALYs rate for (**A)** depressive disorders, (**B**) major depressive disorder, (**C) **bipolar disorder and** (D) **anxiety disorder in both sexes in the world and MENA countries in 2019

 In 2019, the age trend of the DALYs rate of depressive disorders in the countries of the MENA region was higher than the global average. Most DALYs, similar to the global trend, have been increased until the age of 69 years, then, it had a descending trend Figure 1A.

 The depressive disorders-related DALYs and the MDD-related DALYs in Palestine in all age groups were found to be higher than in the MENA region (Figure 1A and 1B). The bipolar disorder-related DALYs in Lebanon in all age groups were found to be higher than in the MENA region (Figure 1C). The anxiety disorder-related DALYs in Iran in all age groups were found to be higher than in the MENA region (Figure 1D).

 The depressive disorders-related DALYs, MDD-related DALYs, and dysthymia disorder-related DALYs have been increased from 15 to 69 years, then, it had a descending trend (Figure 1A and 1B). Also, bipolar disorder-related DALYs have been increased from 15 to 49 years, then, it had a descending trend (Figure 1C). Anxiety disorder-related DALYs have been increased from 4 to 49 years, then, it had a descending trend (Figure 1D).

**Figure 2:**
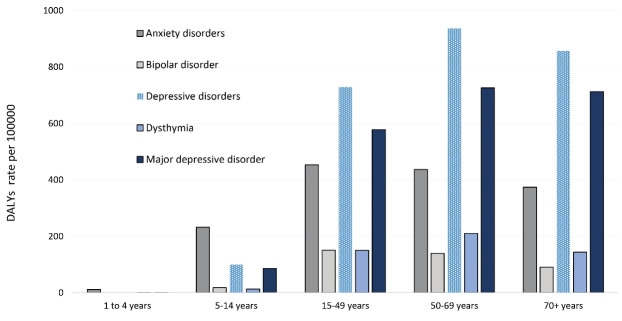
MENA DALYs rate of depressive, bipolar, anxiety, dysthymia and major depressive disorder in both sexes in the world in 2019, by age

 Except for the age group under 15 years, the DALYs rate of depression disorder was higher than other disorders in all age groups. The DALYs rate of depression, MDD, and dysthymia disorders in the age group of 50-69 years was higher than other age groups. The DALYs rate of bipolar and anxiety disorders in the age group of 15-49 years was higher than other age groups. The burden of anxiety disorders in the age group of 1-15 years was higher than other disorders Figure 2.

**Figure 3:**
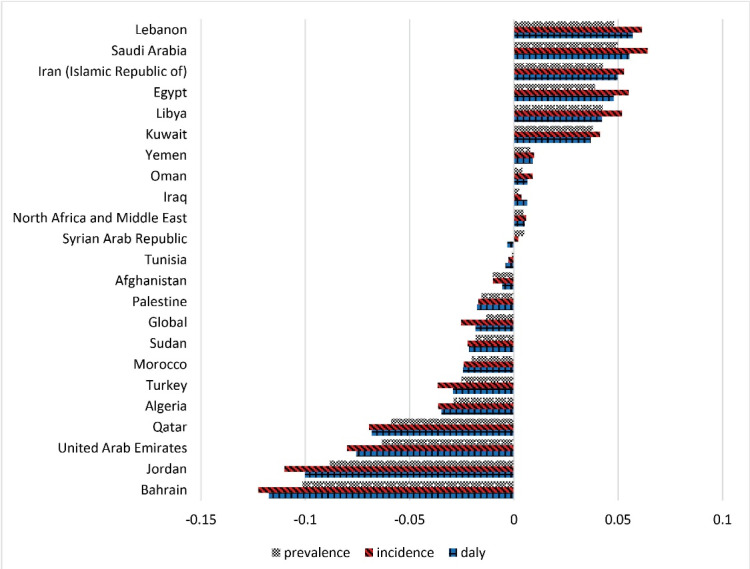
Percentage of changes in depressive disorder by ASR in both sex from 1990 to 2019

 Between 1990 and 2019, the highest decrement of the depressive disorders-related prevalence, incidence, and DALYs rates was related to Bahrain by 0.101%, 0.122%, and 0.117%. Moreover, the highest increment of the depressive disorders-related prevalence, incidence, and DALYs rates was related to Saudi Arabia by 0.05%, 0.064%, and 0.055%, and also Lebanon by 0.048%, 0.061%, and 0.057%. The depressive disorders-related prevalence, incidence, and DALYs rates in the MENA region increased by 0.004%, 0.006%, and 0.005%. The global prevalence, incidence, and DALYs rates of depressive disorders decreased by 0.013%, 0.025%, and 0.018% Figure 3.

**Figure 4:**
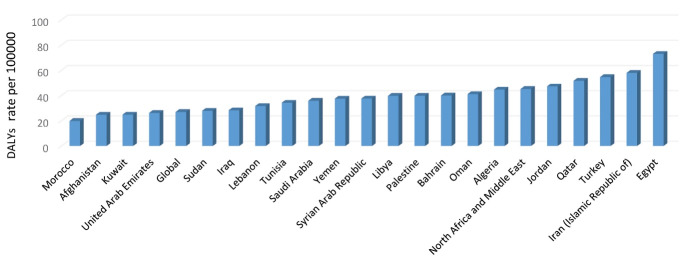
The DALYs rate of anxiety disorder attributable to Bullying victimization in 2019

 The highest percentage of anxiety disorder-related DALYs attributed to bullying victimization was related to Egypt (72.99), Iran (57.93) and Turkey (54.61), and Morocco (19.82) had the lowest anxiety disorder-related DALYs due to bullying victimization Figure 4.

**Figure 5:**
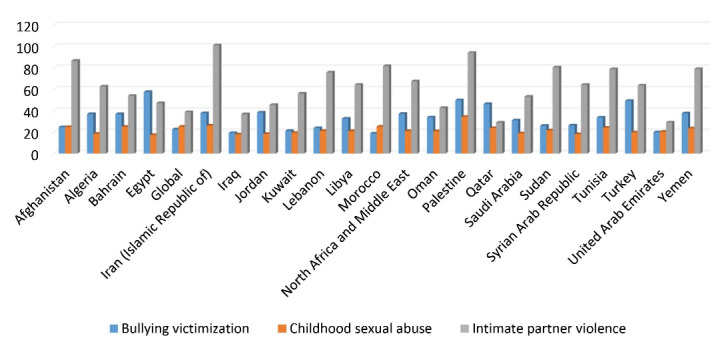
The DALYs rate of major depression disorder attributable to bullying victimization, childhood sexual abuse and intimate partner violence in 2019

 The highest percentage of MDD-related DALYs attributed to intimate partner violence was related to Iran (101.1), Palestine (94.4), and Afghanistan (86.62). The rate of MDD-related DALYs attributed to intimate partner violence was 38.65 in the world and 67.36 in the MENA region. The highest percentage of MDD-related DALYs attributed to childhood sexual abuse was related to Palestine (34.26). The rate of MDD-related DALYs attributed to childhood sexual abuse was 25.11 in the world and 21.04 in the MENA region. The highest percentage of MDD-related DALYs attributed to bullying victimization was related to Palestine (49.81), Turkey (49.14), and Qatar (46.18). The rate of MDD-related DALYs attributed to bullying victimization was 22.46 in the world and 36.97 in the MENA region Figure 5.

## Discussion

 Depressive disorders are a major public health problem and a leading cause of disability. The present study used data published in the GBD database to determine the epidemiological features and the burden of depressive disorders in the MENA region from 1990 to 2019. The results of this study can be used by governments in MENA regions to develop beneficial policies for preventing depressive disorders.

 This study found that the depressive disorders-related prevalence, incidence, and DALYs rates in MENA increased by 0.004%, 0.006%, and 0.005% from 1990 to 2019. This may be due to that North Africa/Middle East includes conflict countries such as Afghanistan, Iraq, and Lebanon. This shows the importance of future mental health research in conflict countries. Based on the evidence, the prevalence of MDD and anxiety disorders was highest in conflict countries with a history of war, many of which are in North Africa and the Middle East.^21-23^ This is in line with literature indicating that exposure to torture and trauma in conflict status growth the prevalence of depressive disorders and post-traumatic stress and anxiety disorders.^24^

 The prevalence, incidence, and DALYs by ASR were highest in Palestine, which is a poor country located in Western Asia that is involved in civil war, dispersion and displacement, tensions, political conflicts. In addition, the depressive disorders-related DALYs and the MDD-related DALYs in Palestine in all age groups were found to be higher than in the MENA region. The Palestinian people are facing extremely difficult situations and limited resources. Furthermore, the daily physical and psychological pressure from political oppression is resulting in confusion and stress. Therefore, the person is unable to achieve his/her normal social status, feeling useless, Isolated, abused, and finally depressed.^25^ These characteristics of the situation in Palestine mean that maintain peace, security, human rights, social liberation and good governance could be a key measure to control depressive disorders.^26^

 Between 1990 and 2019, the depressive disorders-related prevalence, incidence, and DALYs increased the most in Saudi Arabia, followed by Lebanon. More research is needed to understand the causes of increased prevalence, incidence, and DALYs in depressive disorders in these countries. Moreover, the bipolar disorder-related DALYs in Lebanon in all age groups were found to be higher than in the MENA region. As a result, more attention must be given to mental health, because there are more people living with depressive disorders and the tendency is for it to increase as the population ages. Especially in less developed countries such as Saudi Arabia and Lebanon, the life expectancy has been increased due to control of infectious diseases, improve reproductive health, nutrition, so results in more people living until adulthood, the mean age of population increases, and the burden of disease shifts to non-communicable diseases such as depression.^27^ The rapid increases in the prevalence, incidence, and DALYs of depressive disorders in Lebanon could be due to civil wars and political conflicts.

 The country with the largest reductions in depressive disorders-related prevalence, incidence, and DALYs was Bahrain. This indicates that Bahrain pays greater attention to this problem in the last decades and should continue to take measures aimed at controlling depressive disorders.

 Furthermore, the anxiety disorder-related DALYs in Iran in all age groups were found to be higher than in the MENA region, mostly due to stress, the lack of employment, and low income. Iran is now experiencing one of the biggest economic crises in its history, with high inflation and millions unemployed, which is probably affect the scenario of the burden of depressive disorders in the coming years.

 The present study showed that there are high rates of depressive disorders in some countries and also depressive disorders especially increased the most in some regions, presenting the importance of identifying the underlying reasons. The pathogenesis of depressive disorders is unclear, however, evidence has determined risk factors for depressive disorders. Research has shown that a genetic predisposition is also linked to depressive disorders, the risk of depressive disorders is considerably higher in first-degree relatives of depressed people than others.^28^ Likewise, women and older people are more likely to suffer from depressive disorders.^29^ Unhealthy habits such as smoking and alcohol consumption increase the risk of depression.^30^ Insomnia increases the risks of depressive disorders.^31^ Depressive disorders are one of the most common neuropsychiatric consequences of cancer, stroke, and AIDS.^32-34^

 Based on the evidence,^35-37^ childhood sexual abuse, intimate partner violence, and bullying victimization have increased the burden of depressive disorders in both sexes. The highest percentage of anxiety disorder-related DALYs attributed to bullying victimization was related to Egypt, Iran, and Turkey. The highest percentage of MDD-related DALYs attributed to intimate partner violence was related to Iran. The highest percentage of MDD-related DALYs attributed to childhood sexual abuse and bullying victimization was related to Palestine.

###  Strengths and limitations of the study

 All limitations of the GBD study have been described in detail elsewhere. Briefly, we describe specific limitations. First, DSM-IV and ICD-10 criteria for depressive disorders may not be applicable across all cultural groups in MENA countries as people prefer not to reveal mental health due to stigma and discrimination. Second, another limitation of this study is a lack of information on the other risk factors. Third, the GBD study used disability weights to compute health loss whereas the impact of depressive disorders on economic productivity and efficiency, and socio-emotional interaction, were not calculated. fourth, the lack of assessment of YLLs makes challenges in the full estimation of health loss due as they are not coded as causes of death in the GBD study based on the ICD-10 criteria, also, indirect mortality attributable to depressive disorders e.g. suicide has been ignored. In July 2011, South Sudan gained independence and separated from Sudan but in this study, data for Sudan and South Sudan are reported as one country. Furthermore, the quality of the data collection system is different across countries, so, the lack of high-quality epidemiological data for all countries is one of the limitations of the GBD study. This study has some strengths. It compared the data of countries that have the same information registration system and socio-demographic Index. Other strengths of the present study were the comprehensive estimations of depressive disorders burden as reported by prevalence, incidence, and DALYs between different countries from 1990 to 2019, hence, it can detect the strengths and weaknesses of the health care systems in different countries.

## Conclusion

 The study indicates that depressive disorders have continued to increase in prevalence, incidence, and DALYs rates in the MENA region, and Palestine has the highest prevalence, incidence, and DALYs by ASR. This increasing trend may be attributed to that North Africa/Middle East includes conflict countries with a history of war. Therefore, mental health services and programs should be prioritized across the MENA countries, with significant contributions and involvement of the governments to improve population mental health and well-being.

## Acknowledgments

 We would like to acknowledge all the authors of the reviewedpublications.

## Author Contributions

**Data curation: **Mehdi Moradinazar.

**Formal Analysis: **Mehdi Moradinazar.

**Funding acquisition: **Mehdi Moradinazar.

**Investigation:** Mona Basiri.

**Methodology: **Mehdi Moradinazar.

**Project administration: **Mehdi Moradinazar.

**Resources: **Mehdi Moradinazar.

**Software: **Mahdieh Saeedi.

**Supervision: **Mohammad Shakiba.

**Validation: **Mahdieh Saeedi.

**Visualization: **Mohammad Shakiba.

**Writing – original draft: **Mohammad Shakiba.

**Writing – review & editing: **Parmida Mirzaie, Mona Basiri.

## Funding

 No found.

## Ethical Approval

 Ethics approval and informed consent were not needed for the present study because of public availability to the data of GBD.

## Competing Interests

 The authors declared no potential conflicts of interest with respect to the research, authorship, and/or publication of this article.

## References

[1] Vos T, Abajobir AA, Abate KH, Abbafati C, Abbas KM, Abd-Allah F (2017). Global, regional, and national incidence, prevalence, and years lived with disability for 328 diseases and injuries for 195 countries, 1990-2016: a systematic analysis for the Global Burden of Disease Study 2016. Lancet.

[2] Afshin A, Sur PJ, Fay KA, Cornaby L, Ferrara G, Salama JS (2019). Health effects of dietary risks in 195 countries, 1990-2017: a systematic analysis for the Global Burden of Disease Study 2017. Lancet.

[3] Yang L, Zhao Y, Wang Y, Liu L, Zhang X, Li B (2015). The effects of psychological stress on depression. Curr Neuropharmacol.

[4] Moussavi S, Chatterji S, Verdes E, Tandon A, Patel V, Ustun B (2007). Depression, chronic diseases, and decrements in health: results from the World Health Surveys. Lancet.

[5] Eastwood J, Ogbo FA, Hendry A, Noble J, Page A (2017). The impact of antenatal depression on perinatal outcomes in Australian women. PLoS One.

[6] Ogbo FA, Eastwood J, Hendry A, Jalaludin B, Agho KE, Barnett B (2018). Determinants of antenatal depression and postnatal depression in Australia. BMC Psychiatry.

[7] Mackenzie S, Wiegel JR, Mundt M, Brown D, Saewyc E, Heiligenstein E (2011). Depression and suicide ideation among students accessing campus health care. Am J Orthopsychiatry.

[8] Luo Y, Zhang S, Zheng R, Xu L, Wu J (2018). Effects of depression on heart rate variability in elderly patients with stable coronary artery disease. J Evid Based Med.

[9] Seligman F, Nemeroff CB (2015). The interface of depression and cardiovascular disease: therapeutic implications. Ann N Y Acad Sci.

[10] Barry MM (2009). Addressing the determinants of positive mental health: concepts, evidence and practice. Int J Ment Health Promot.

[11] Lozano R, Fullman N, Mumford JE, Knight M, Barthelemy CM, Abbafati C (2020). Measuring universal health coverage based on an index of effective coverage of health services in 204 countries and territories, 1990-2019: a systematic analysis for the Global Burden of Disease Study 2019. Lancet.

[12] James SL, Abate D, Abate KH, Abay SM, Abbafati C, Abbasi N (2018). Global, regional, and national incidence, prevalence, and years lived with disability for 354 diseases and injuries for 195 countries and territories, 1990-2017: a systematic analysis for the Global Burden of Disease Study 2017. Lancet.

[13] Murray CJ, Aravkin AY, Zheng P, Abbafati C, Abbas KM, Abbasi-Kangevari M (2020). Global burden of 87 risk factors in 204 countries and territories, 1990-2019: a systematic analysis for the Global Burden of Disease Study 2019. Lancet.

[14] Hay SI, Abajobir AA, Abate KH, Abbafati C, Abbas KM, Abd-Allah F (2017). Global, regional, and national disability-adjusted life-years (DALYs) for 333 diseases and injuries and healthy life expectancy (HALE) for 195 countries and territories, 1990-2016: a systematic analysis for the Global Burden of Disease Study 2016. Lancet.

[15] American Psychiatric Association (APA). Diagnostic and Statistical Manual of Mental Disorders. Washington, DC: APA; 1980.

[16] World Health Organization (WHO). The ICD-10 Classification of Mental and Behavioural Disorders: Clinical Descriptions and Diagnostic Guidelines. Geneva: WHO; 1992.

[17] Lozano R, Naghavi M, Foreman K, Lim S, Shibuya K, Aboyans V (2012). Global and regional mortality from 235 causes of death for 20 age groups in 1990 and 2010: a systematic analysis for the Global Burden of Disease Study 2010. Lancet.

[18] Haagsma JA, Graetz N, Bolliger I, Naghavi M, Higashi H, Mullany EC (2016). The global burden of injury: incidence, mortality, disability-adjusted life years and time trends from the Global Burden of Disease study 2013. Inj Prev.

[19] Ahmad OB, Boschi-Pinto C, Lopez AD, Murray CJ, Lozano R, Inoue M (2001). Age Standardization of Rates: A New WHO Standard.

[20] Porta M (2014). A Dictionary of Epidemiology.

[21] Degenhardt L, Baxter AJ, Lee YY, Hall W, Sara GE, Johns N (2014). The global epidemiology and burden of psychostimulant dependence: findings from the Global Burden of Disease Study 2010. Drug Alcohol Depend.

[22] Ferrari AJ, Charlson FJ, Norman RE, Patten SB, Freedman G, Murray CJ (2013). Burden of depressive disorders by country, sex, age, and year: findings from the Global Burden of Disease Study 2010. PLoS Med.

[23] Whiteford HA, Ferrari AJ, Degenhardt L, Feigin V, Vos T (2015). The global burden of mental, neurological and substance use disorders: an analysis from the Global Burden of Disease Study 2010. PLoS One.

[24] Steel Z, Chey T, Silove D, Marnane C, Bryant RA, van Ommeren M (2009). Association of torture and other potentially traumatic events with mental health outcomes among populations exposed to mass conflict and displacement: a systematic review and meta-analysis. JAMA.

[25] Qouta S. Trauma, Violence, and Mental Health: The Palestinian Experience. S.R.I. Quta; 2000.

[26] Jabr S, Morse M, El Sarraj W, Awidi B (2013). Mental health in Palestine: country report. Arab J Psychiatr.

[27] McKee S. Rethinking Development and Health: Findings from the Global Burden of Disease Study. Institute for Health Metrics and Evaluation; 2016.

[28] Mullins N, Lewis CM (2017). Genetics of depression: progress at last. Curr Psychiatry Rep.

[29] Zhao L, Han G, Zhao Y, Jin Y, Ge T, Yang W (2020). Gender differences in depression: evidence from genetics. Front Genet.

[30] Gravely-Witte S, Stewart DE, Suskin N, Grace SL (2009). The association among depressive symptoms, smoking status and antidepressant use in cardiac outpatients. J Behav Med.

[31] Li L, Wu C, Gan Y, Qu X, Lu Z (2016). Insomnia and the risk of depression: a meta-analysis of prospective cohort studies. BMC Psychiatry.

[32] Elbadawi A, Mirghani H (2017). Depression among HIV/AIDS Sudanese patients: a cross-sectional analytic study. Pan Afr Med J.

[33] Sotelo JL, Musselman D, Nemeroff C (2014). The biology of depression in cancer and the relationship between depression and cancer progression. Int Rev Psychiatry.

[34] Hackett ML, Yapa C, Parag V, Anderson CS (2005). Frequency of depression after stroke: a systematic review of observational studies. Stroke.

[35] Sagar R, Dandona R, Gururaj G, Dhaliwal R, Singh A, Ferrari A (2020). The burden of mental disorders across the states of India: the Global Burden of Disease Study 1990-2017. Lancet Psychiatry.

[36] Bauer NS, Herrenkohl TI, Lozano P, Rivara FP, Hill KG, Hawkins JD (2006). Childhood bullying involvement and exposure to intimate partner violence. Pediatrics.

[37] Bhatta DK, Budhathoki K, Paudel K, Paudel S, Marhatta SB, Banskota A, et al. Burden of depressive and anxiety disorders in Nepal, 1990-2017: an analysis of global burden of disease data. medRxiv [Preprint]. June 15, 2021. Available from: https://www.medrxiv.org/content/10.1101/2021.06.13.21258841v1.

